# A Novel Constrained Non-negative Matrix Factorization Method for Group Functional Magnetic Resonance Imaging Data Analysis of Adult Attention-Deficit/Hyperactivity Disorder

**DOI:** 10.3389/fnins.2022.756938

**Published:** 2022-02-17

**Authors:** Ying Li, Weiming Zeng, Yuhu Shi, Jin Deng, Weifang Nie, Sizhe Luo, Jiajun Yang

**Affiliations:** ^1^Lab of Digital Image and Intelligent Computation, Shanghai Maritime University, Shanghai, China; ^2^College of Mathematics and Information, South China Agricultural University, Guangzhou, China; ^3^Department of Neurology, Shanghai Jiao Tong University Affiliated Sixth People’s Hospital, Shanghai, China

**Keywords:** attention deficit hyperactivity disorder, constrained non-negative matrix factorization, dynamic functional connectivity, functional magnetic resonance imaging, intrinsic reference

## Abstract

Attention-deficit/hyperactivity disorder (ADHD) is a common childhood psychiatric disorder that often persists into adulthood. Extracting brain networks from functional magnetic resonance imaging (fMRI) data can help explore neurocognitive disorders in adult ADHD. However, there is still a lack of effective methods to extract large-scale brain networks to identify disease-related brain network changes. Hence, this study proposed a spatial constrained non-negative matrix factorization (SCNMF) method based on the fMRI real reference signal. First, non-negative matrix factorization analysis was carried out on each subject to select the brain network components of interest. Subsequently, the available spatial prior information was mined by integrating the interested components of all subjects. This prior constraint was then incorporated into the NMF objective function to improve its efficiency. For the sake of verifying the effectiveness and feasibility of the proposed method, we quantitatively compared the SCNMF method with other classical algorithms and applied it to the dynamic functional connectivity analysis framework. The algorithm successfully extracted ten resting-state brain functional networks from fMRI data of adult ADHD and healthy controls and found large-scale brain network changes in adult ADHD patients, such as enhanced connectivity between executive control network and right frontoparietal network. In addition, we found that older ADHD spent more time in the pattern of relatively weak connectivity. These findings indicate that the method can effectively extract large-scale functional networks and provide new insights into understanding the neurobiological mechanisms of adult ADHD from the perspective of brain networks.

## Introduction

Attention-deficit/hyperactivity disorder (ADHD) is a mental disorder portrayed by inattention, hyperactivity, and impulsivity. Studies had shown that approximately 5% of school-age children have ADHD, and symptoms can last to adulthood (Kooij et al., 2010). Considering that children with ADHD are not sensitive to their mental state, it is easier to explore the mystery of ADHD in adults than in children. At present, the etiology and pathogenesis of adult ADHD are not well understood, but it is certain that adult ADHD is inextricably related to the disorder of brain cognitive neural network connectivity. Generally, a healthy brain can be described as an optimized network organization, which is composed of spatially separated brain functional networks with dense functional connectivities (FCs) within brain networks and sparse FCs between brain networks. Brain functional networks play a crucial role in maintaining the balance between functional specialization and integration, supporting individual cognitive, and behavioral abilities (He et al., 2009; Crossley et al., 2013; Bertolero et al., 2015; Sporns and Betzel, 2016). For example, studies had confirmed that the intra-network and inter-network FCs of the frontal parietal network are disrupted in patients with ADHD (Cortese et al., 2012). Accordingly, it is necessary to study ADHD’s brain dysfunction network connection in order to identify biomarkers associated with cognitive impairment in patients for clinical diagnosis and optimization of disease treatment, while helping to understand and identify the neurobiological mechanisms of ADHD.

Recent advances had manifested that the combination of resting-state functional magnetic resonance imaging (fMRI) technique and FC analysis framework can help elucidate the disruption of internal brain functional networks in patients with psychiatric disorders (Buckholtz and Meyer-Lindenberg, 2012; Bertolero et al., 2015; Gong et al., 2017). The calculation of FC from the fMRI time series, particularly in the course of the resting state, has manifested a mountain of understanding and awareness concerning the macroscopic spatiotemporal organization of the brain. Moreover, FC within the brain probably varied by psychotic disorders or in the course of evolutionary phases. The secondary analysis of Castellanos et al. (2008) revealed an ADHD-related FC descent pattern between the precuneus and other default mode network components. In addition, previous studies had proved that FC between brain networks changed with increasing age (Allen et al., 2011; Qin et al., 2015). The FC mentioned above is commonly referred to as the static FC approach, assuming that neural activity between brain regions remains stationary throughout the fMRI scan (Calhoun et al., 2014). However, this assumption of stability may not hold because there are no restrictions on brain activity in resting states, and spontaneous fluctuations in brain activity are significant. Taking this property into account, dynamic functional connectivity approaches have been proposed, which can provide unique temporal information (Allen et al., 2014). In addition, brain networks have characteristics that change over time. Hence, this study uses dynamic FC to delve into the interactions within and between large-scale brain networks of ADHD over a period of time, which facilitates the discovery of reproducible patterns of brain functional network impairment and reveals connectivity differences between ADHD patients and healthy control (HC) subjects.

At present, there are many methods to extract brain function networks and their corresponding time series from resting-state fMRI data, such as non-negative matrix decomposition (NMF) and independent component analysis (ICA) (Calhoun et al., 2003; Wang et al., 2004). ICA is a holistic decomposition, where each base is statistically assumed to be independent (Shi et al., 2017, 2018b). However, the presence of multiple zero components in the fMRI data makes it difficult to handle higher-order averages and cannot effectively extract sparse sources. NMF can effectively solve this problem. NMF is a practicable data-driven multi-analysis method that breaks down non-negative data sets into sparse, partially based linear combinations of non-negative features (Deng et al., 2018). In addition, NMF is a nimble approach, which can be developed into a constrained NMF method by adding different constraints to the estimation process. Compared with the classic NMF method, constrained NMF only extracts the expected components by adding prior information to the algorithm, which is beneficial to the subsequent application and reduces the calculation time and storage requirements in the process of NMF analysis. However, the prior information contained in the current constrained NMF method is mostly some specific knowledge about the source signal, such as the experimental paradigm in specific cognitive tasks (Ferdowsi et al., 2010), which does not exist in most cases. Therefore, it is worth considering how to pick up the available authentic information from fMRI data. A recently published article by Shi et al. (2015) put forward a method called group ICA with the intrinsic reference, which extracts the procurable prior message from the group data. Inspired by this method, we presented a new spatial constrained non-negative matrix decomposition (SCNMF) approach to obtain *a priori* information by extracting available real information from the group fMRI data and integrate this *a priori* information into the estimation process of the NMF method.

Generally speaking, NMF is mostly used to analyze fMRI data of a single subject, but in practical application, we need to analyze multiple subjects’ fMRI data in more cases. According to the different hypothesis conditions for multi-subjects, there are different processing methods for multi-subject fMRI data analysis, containing spatial concatenation, temporal concatenation, and tensor concatenation. Supposing that total subjects have the same temporal model message, and the fMRI data of multiple subjects are connected according to the spatial dimension, this method is called the spatial concatenation method (Svensén et al., 2002). Supposing that all subjects have the same spatial model message, and the fMRI data of multiple subjects are connected according to the temporal dimension, this method is called the temporal concatenation method (Calhoun et al., 2002; Beckmann et al., 2009). Supposing that all subjects have the same spatial and temporal pattern message except for the different magnitude, and the fMRI data of multiple subjects are connected according to a separate third dimension, this method is called the tensor concatenation method (Beckmann and Smith, 2005; Lee et al., 2008). Previous studies have enunciated that the temporal concatenation method has a better fMRI group analysis function in these three concatenation methods (Schmithorst and Holland, 2004; Guo and Pagnoni, 2008). Therefore, this manuscript applies the time concatenation way to fMRI data on multiple subjects. At the level of multi-subject, it is simply concatenation according to temporal, which has the problems of a large amount of data sample and high time complexity. To solve this problem, we first reduced the data, then conducted SCNMF analysis on the reduced data, and then reversed reconstruction to obtain the brain functional network information of each subject and its corresponding time process.

In conclusion, in order to better study brain network disorders in adult ADHD patients, we developed a new SCNMF method to extract large-scale functional networks. The proposed SCNMF method was quantitatively compared with two classical methods, including NMF and FastICA. Subsequently, we presented a new analysis framework to evaluate the variations of large-scale functional networks in adult ADHD and HCs. Specifically, the SCNMF method proposed in this manuscript is used to extract the brain function network information of each subject and its corresponding time process, and then dynamic FC and clustering were performed on the time series of all subjects to get the dynamic functional states. The framework calculates group-level connection states by analyzing the dynamic FCs of subjects within a group and then estimates mutually independent subject-specific connection states accordingly, guided by the group-level states. Thus, the resulting subject-specific states capture both inter-subject variability and intra-group similarity. In addition, we used univariate analysis to explore the effect of age on dynamic functional states in adult ADHD and HCs.

## Materials and Methods

### Participants

In this study, the resting fMRI data of 25 adult ADHD and 24 age-matched HCs were obtained from the open database.^1^ Demographic characteristics are shown in Table 1. The patients and HCs were recruited by the adult ADHD program of the University of New York School of Medicine, whose data collection process and parameters were described as follows: the patient lay flat in the MRI, opened his eyes, remained relaxed, and awoke. The scanning repetition time TR was 2,000 ms, echo time TE was 25 ms, the scanning resolution was 64 × 64, the chip resolution was 3 mm × 3 mm, the slice thickness was 3 mm, the number of slices was 39 slices, covering the whole brain area, and the acquisition consists of 192 time points.

**TABLE 1:** Demographic characteristics.

	ADHD	HC
Age (mean ± SD)	34.75 ± 9.71	34.8 ± 7.99
Sex (male/female)	19/5	15/9

### Data Preprocessing

To stabilize the magnetic field of the scanning machine and adapt the subjects to the scanning environment, the first ten time points of the fMRI image were abandoned. The next preprocessing procedures were slice timing and head motion realign. During head motion realign, one ADHD subject was excluded due to excessive head movement, based on a criterion of 3 mm or 3°. Next, the images were registered to the standard template of the Montreal Institute of Neurology (MNI) and then resampled to 2 mm isotropic resolution. Spatial smoothing with a full-width Gaussian core at half-maximum (FWHM) of 6 mm was used to improve the signal-to-noise ratio (SNR) of fMRI data. To reduce noise caused by head movements, the covariate Friston-24 head movement parameter was regressed from the BOLD time series of all voxels.

### Non-negative Matrix Factorization

Given the matrix $X\in R_{+}^{T\times M}$, find the non-negative matrix $W\in R_{+}^{T\times K}$ and the non-negative matrix $H\in R_{+}^{K\times M}$, so that:


(1) $$\begin{array}{c} X_{T\times M}\approx W_{T\times K}\,H_{K\times M} \end{array}$$

It is approximate because the current solution is not an exact solution, but only a numerical approximation. *K* is the number of components in the decomposition. The inequality (*T* + *M*)*K* < *T*×*M* represents that only a small number of bases is used to describe a large amount of data. Therefore, it is possible to make *X=WH* only if *W* contains the intrinsic characteristics of the random variable.

In addition, NMF is often interpreted by scholars with the background of blind signal separation as a generation model with noise-containing items (Cichocki et al., 2006), which is defined as:


(2) $$\begin{array}{c} X=W\,H+\varepsilon \end{array}$$

ε is the noise matrix of *T*×*M*. The different NMF algorithms are interpreted as complying with the maximum likelihood algorithm under different ε distribution hypotheses. The process of realizing NMF is a process of optimization solution. Donoho and Stodden (2004) analyzed the requirements for the presence of a unique solution to NMF theoretically. The harshness of this condition revealed that it is a feasible method to construct a reasonable objective function and then optimize *W* and *H* alternately to obtain a local optimal solution of NMF. The classic method to seek *W* and *H* is by minimizing the discrepancy among *X* and *WH*, which is defined as:


(3) $$\begin{array}{c} \min_{W\in R_{+}^{S\times K},H\in R_{+}^{K\times M}}J\,\left(W,H\right)={||\text{X–WH}||}_{F}^{2} \end{array}$$

where ||⋅||*_F_* is the Frobenius norm. The matrices *W* and *H* are randomly initialized, and then the Euclidean distance function is minimized by updating *W* and *H* alternately. The multiplication update rules [30] for *W* and *H* are as follows:


(4) $$\begin{array}{c} W_{i\,j}\leftarrow W_{i\,j}\,\frac{{\left(X\,H^{\text{T}}\right)}_{i\,j}}{{\left(W\,H\,H^{\text{T}}\right)}_{i\,j}} \end{array}$$

(5) $$\begin{array}{c} H_{i\,j}\leftarrow H_{i\,j}\,\frac{{\left(W^{\text{T}}\,X\right)}_{i\,j}}{{\left(W^{\text{T}}\,W\,H\right)}_{i\,j}} \end{array}$$

### Extraction of Intrinsic Prior Information

**FIGURE 1:**
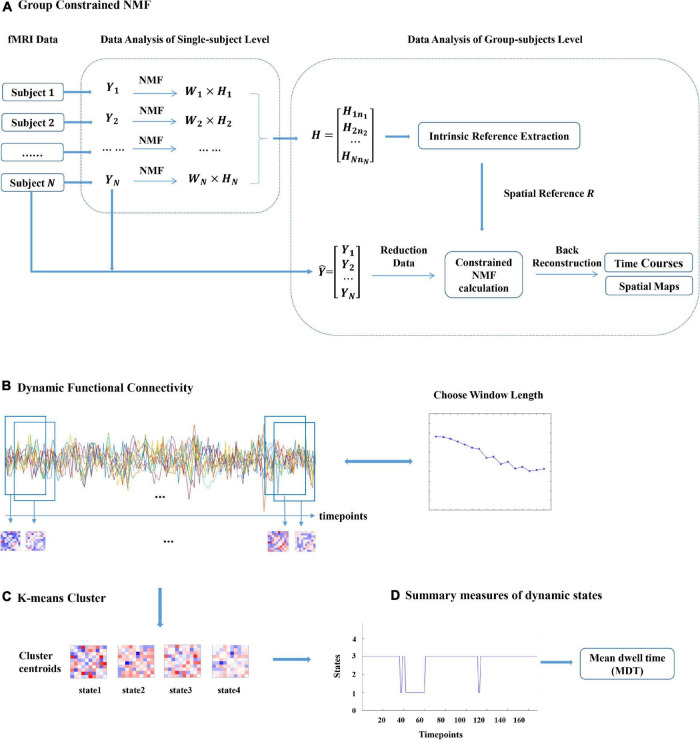
Graphical description of the analysis approach. **(A)** *X*_*i*_(*i* = 1,2,…,*N*) denotes the functional magnetic resonance imaging (fMRI) data of subject *i*. *W*_*i*_ and *H*_*i*_ denote the temporal and spatial components of subject *i* gained by non-negative matrix decomposition (NMF). *R* denotes the spatial prior information obtained from *H* by the proposed NMF with the intrinsic reference method and incorporates this prior information into the new constrained NMF method. The proposed SCNMF method for the group fMRI analysis is composed of data reduction, constrained NMF calculation, and back reconstruction. **(B)** The sliding window approach is adopted to construct a dynamic FC matrix between each of the selected subject’s component. **(C)** K-means clustering is applied to identify dynamic FC states. **(D)** Summary measures of dynamic states, such as the mean dwell time (MDT) and the fraction of time (FT).

Inspired by the group ICA with intrinsic reference proposed by Shi et al. (2015), we presented an NMF with intrinsic reference method based on the intrinsic reference signal to guide the NMF analysis of fMRI data by mining the available prior information from multiple subject fMRI data. The general process framework for this method is shown in Figure 1A.

Assume that there are *N* subjects in a group fMRI data set, and each subject after normalization has *T* time points and *M* voxels. First, NMF analysis is performed separately for each subject, and for subject *i*, NMF is expressed as:


(6) $$\begin{array}{c} X_{i}=W_{i}H_{i}\left(i=1,2,\ldots ,N\right) \end{array}$$

Where *X*_*i*_ represents the *T*×*M* fMRI observation data, *W*_*i*_ represents the *T*×*K*_*i*_ mixing matrix, and each row of $H_{i}={\left(H_{i}^{1},H_{i}^{2},\ldots ,H_{i}^{k_{i}}\right)}^{\text{T}}$ represents a component of subject *i* after NMF.

**FIGURE 2:**
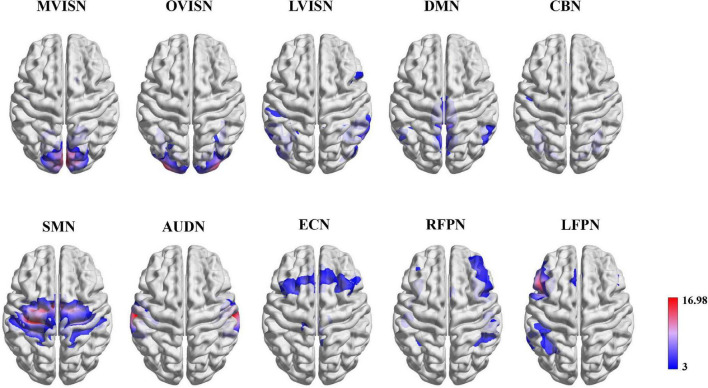
Functional network maps. The functional network z-score maps defined by the ten resting-state functional networks.

We selected ten resting-state functional network templates extracted from the BrainMap database (Smith et al., 2009) as the region of interest (ROI), including the medial visual network (MVISN), occipital pole visual network (OVISN), lateral visual network (LVISN), default mode network (DMN), cerebellum network (CBN), sensorimotor network (SMN), auditory network (AUDN), executive control network (ECN), right frontoparietal network (RFPN), and left frontoparietal network (LFPN). The visualization of the ten resting-state functional networks is shown in Figure 2. According to the correlation coefficient between the network template and the components of each subject, the component with the maximum correlation coefficient was selected and denoted as $H_{i}^{k_{i}}\left(i=1,2,\ldots ,N\right)$ (6)

Subsequently, we extracted the spatial prior information from $H_{i}^{k_{i}}\left(i=1,2,\ldots ,N\right)$ by principal component analysis method to reduce the impact of noise (Langley et al., 2010). All $H_{i}^{k_{i}}\left(i=1,2,\ldots ,N\right)$ can form a matrix of *N*×*M*, namely,


(7) $$\begin{array}{c} \bar{H}={\left(H_{1}^{k_{1}},H_{2}^{k_{2}},\ldots ,H_{N}^{k_{N}}\right)}^{\text{T}} \end{array}$$

Whereafter, the eigenvalues and eigenvectors of the covariance matrices of $\bar{H}$ can be calculated. The eigenvector of $\bar{H}$ is expressed as *e*_*n*_(*n* = 1,2,…,*N*). Furthermore, the first principal component $R=e_{1}^{T}\,\bar{H}$ can be obtained, which is the implicit spatial prior information related to the component of interest in the fMRI data of the subjects in the group, where *e*_*1*_ represents the feature vector corresponding to the maximum eigenvalue.

### Spatial Constrained Non-negative Matrix Factorization

When the prior information is obtained by the above method, we formulate a spatial constrained NMF method containing spatial prior information grounded on the multi-objective optimization framework as follows:


(8) $$\begin{array}{c} \begin{array}{c} M\,i\,n\,i\,m\,i\,z\,e\,\left\{ \begin{array}{c} J\,\left(W,H\right)={||\text{X–WH}||}_{F}^{2}\,\\ \varepsilon \,\left(H\right)={||\text{H–R}||}_{F}^{2}\, \end{array}\right.\\ S\,u\,b\,j\,e\,c\,t\,\,t\,o\,\,W\geq 0,\,H\geq 0 \end{array} \end{array}$$

where *R* represents the spatial prior signal and $\varepsilon \,\left(H\right)={||\text{H-R}||}_{F}^{2}$ is used to measure the similarity between *H* and *R*.

Therefore, the objective function *f*(*W*,*H*) can be redefined as follows:


(9) $$\begin{array}{c} f\,\left(W,H\right)=a\cdot J\,\left(W,H\right)+b\cdot \varepsilon \,\left(H\right) \end{array}$$

where *a* and *b* are the weighting parameters.


(10) $$\begin{array}{c} J\left(W,H\right)=tr\left(X^{\text{T}}X\right)-2tr\left(H^{\text{T}}W^{\text{T}}X\right)++tr\left(H^{\text{T}}W^{\text{T}}WH\right) \end{array}$$

(11) $$\begin{array}{c} \varepsilon \,\left(H\right)=t\,r\,\left(H^{\text{T}}\,H\right)-2\,t\,r\,\left(R^{\text{T}}\,H\right)+t\,r\,\left(R^{\text{T}}\,R\right) \end{array}$$

Let ψ_*ij*_ and φ_*ij*_ be constrained Lagrange multipliers of *W*_*ij*_≥0*andH*_*ij*_≥0:


(12) $$\begin{array}{c} L\,\left(W,H\right)=f\,\left(W,H\right)+t\,r\,\left(\Psi \,W^{\text{T}}\right)+t\,r\,\left(\Phi \,H^{\text{T}}\right) \end{array}$$

where Ψ = [ψ_*ij*_], Φ = [φ_*ij*_]

The partial derivatives of *L*(*W*,*H*) with respect to *W* and *H* are:


(13) $$\begin{array}{c} \frac{\partial L}{\partial W}=-2\,a\cdot X\,H^{\text{T}}+2\,a\cdot W\,H\,H^{\text{T}}+\Psi \end{array}$$

(14) $$\begin{array}{c} \frac{\partial L}{\partial H}=-2\,a\cdot W^{\text{T}}\,X+2\,a\cdot W^{\text{T}}\,W\,H+2\,b\cdot H-2\,b\cdot R+\Phi \end{array}$$

Based on the Karush–Kuhn–Tucker (KKT) condition, Ψ_*ij*_*W*_*ij*_ = 0*and* Φ_*ij*_*H*_*ij*_ = 0. We can obtain the equation of *W*_*ij*_ and *H*_*ij*_:


(15) $$\begin{array}{c} {\left(X\,H^{\text{T}}\right)}_{i\,j}\,W_{i\,j}+{\left(W\,H\,H^{\text{T}}\right)}_{i\,j}\,W_{i\,j}=0 \end{array}$$

(16) $$\begin{array}{c} a\cdot {\left(-W^{\text{T}}\,X+W^{\text{T}}\,W\,H\right)}_{i\,j}\,H_{i\,j}+b\cdot {\left(H-R\right)}_{i\,j}\,H_{i\,j}=0 \end{array}$$

Whereafter, the update rules of *W* and *H* are as follows,


(17) $$\begin{array}{c} W_{i\,j}\leftarrow W_{i\,j}\,\frac{{\left(X\,H^{\text{T}}\right)}_{i\,j}}{{\left(W\,H\,H^{\text{T}}\right)}_{i\,j}} \end{array}$$

(18) $$\begin{array}{c} H_{i\,j}\leftarrow H_{i\,j}\,\frac{{\left(a\cdot W^{\text{T}}\,X+b\cdot R\right)}_{i\,j}}{{\left(a\cdot W^{\text{T}}\,W\,H+b\cdot H\right)}_{i\,j}} \end{array}$$

Consequently, *W* and *H* are constantly updated to meet the convergence rule, that is, to reach the comparative error of the set number or the iteration number of the set value.

When the SCNMF algorithm proposed above extracts fMRI data of multiple subjects, there are problems of large data sample size and time redundancy. Therefore, in this study, the fMRI data of a group of subjects were first downscaled, and then SCNMF analysis was performed on the downscaled data, followed by inverse reconstruction to obtain the brain functional network information of each subject and its corresponding time series, as shown in Figure 1A.

Data reduction is to cut down the temporal dimensionality of fMRI data for the benefit of subsequent applications. Two data reduction steps are used for multiple subjects. Supposing that there are *N* subjects in the aggregate, *X*_*i*_ is the *T*×*M* data matrix from subject *i*, *T* is the number of fMRI time points, and *M* is the number of voxels. *K* is the number of estimated components in the temporal dimension. The first step is to perform a PCA descending dimension for each subject, as follows:


(19) $$\begin{array}{c} Y_{i}=F_{i}^{-1}\,X_{i} \end{array}$$

where $F_{i}^{-1}\,$ is the *L*×*T* reducing matrix. After the fMRI data of each subject is reduced, they are concatenated into a matrix according to the temporal dimension and then used in the next data reduction step. Let *Y* denote the *S*×*M* reduced data matrix, where *S* is the temporal dimension after the second dimensionality reduction. Then, the concatenated matrix for *N* subjects can be obtained as follows:


(20) $$\begin{array}{c} Y=G^{-1}\,\left[\begin{array}{c} F_{1}^{-1}\,X_{1}\\ \cdots \\ F_{N}^{-1}\,X_{N} \end{array}\right] \end{array}$$

where *G*^−1^ is a reducing matrix *S*×*LN*.

Subsequently, constrained NMF is carried out to obtain the *S*×*K* non-negative matrix *W* and *K*×*M* non-negative matrix *H*, which is denoted as:


(21) $$\begin{array}{c} Y=W\,H \end{array}$$

Then, to calculate the decomposition components of each subject, back reconstruction needs the results of NMF decomposition and data reduction. Subject-specific spatial maps *H*_*i*_ and time courses *W*_*i*_ are gained employing the spatiotemporal regression back reconstruction approach (Erhardt et al., 2011), namely,


(22) $$\begin{array}{c} W_{i}=X_{i}\,H^{-1} \end{array}$$

(23) $$\begin{array}{c} H_{i}=W_{i}^{-1}\,X_{i} \end{array}$$

The matrix *H*_*i*_ contains the *K* spatial maps and the matrix *W*_*i*_ is the time series matrix of subject *i*, which consists of the time point corresponding to *K* components.

In addition, to evaluate the decomposition performance of the SCNMF method, SCNMF was quantitatively compared with two traditional methods, including NMF and FastICA. Specifically, the brain networks of interest were obtained from fMRI data using the SCNMF, NMF, and FastICA methods, respectively. Subsequently, the correlation analysis of the brain network of interest with ten resting-state network templates was carried out, and the experiment was repeated 20 times.

Immediately afterward, the generalized estimating equation method was used to explore whether the correlation coefficients between brain functional templates and brain network components obtained by the three methods (i.e., SCNMF, NMF, and FastICA) were statistically different. Paired *post hoc* analyses were performed for the three methods with Bonferroni correction for multiple comparisons.

### Dynamic Functional Connectivity Analysis

To verify that the proposed method SCNMF in this manuscript can effectively extract large-scale functional networks associated with cognitive deficits, we applied the SCNMF method to fMRI data of adult ADHD and HCs, and mainly analyzed the dynamic FC between the large-scale networks of the two groups of subjects. The sliding window approach was adopted to construct a dynamic FC matrix between ten networks (see Figure 1B). Starting from the first time point, the time series of brain networks were intercepted with a certain window length in steps of 1 TR. Then Pearson correlation coefficients were calculated for the time series within each window length.

After obtaining the dynamic FC matrices of adult ADHD and HCs, respectively, the combination of these dynamics FC of the two groups was regarded as the cluster samples, and then they were used for k-means clustering (see Figure 1C). However, the traditional k-means algorithm has randomness in centroid initialization. Yao et al. (2013) indicated that automatic target generation processing (ATGP) could effectively obtain the initial mixing matrix in the ATGP-ICA algorithm so that ATGP-ICA has better performance than the traditional random initialization FastICA algorithm in the analysis of fMRI signal. Besides, the sparse dictionary learning separation (SDLS) model proposed by Wang et al. (2016) also manifested that ATGP initialization can promote the convergence speed and correctness of SDLS in the process of sparse dictionary learning. Accordingly, the ATGP algorithm was applied to seek effective initial centroid from dynamic FC matrix to assist the k-means algorithm in achieving stable clustering results. Furthermore, the optimum value of centroid states was taken stock of, applying the elbow criterion defined as the proportion of internal to among cluster distance.

Then, we used the dynamic state summary metrics mean dwell time (MDT) and time fraction (FT) to investigate the time spent by each subject in different states, as shown in Figure 1D (Shi et al., 2018a). Furthermore, while multiple aspects of functional connectivity show potential for clinical application, the utility of brain network status assessment as a reliable tool depends on the ability to interpret abnormal outcomes. Underlying factors, such as age, are expected to have a large impact on functional connectivity (Allen et al., 2011; Rashid et al., 2018). Therefore, we performed the univariate analysis of MDT and FT in each state to investigate the relevance between FC and age in different states. The univariate model was defined as follows:


(24) $$\tilde{K}=D\,\beta$$

where β is the linear regression coefficient and *D* represents the age matrix for all subjects. Here, age is log-transformed.

## Results

### Algorithm Decomposition Performance Analysis

In this study, *W* and *H* are initialized by assigning values to uniform distributions ranging from 0 to 1. By way of verifying the repeatability of the model, the algorithm model was repeatedly run 20 times in this study to calculate the overlap rate of all results. All the elements of the ten brain network components obtained from the decomposition were to do a union operation to obtain a one-dimensional eigenvector.

The overlap rate is defined as:


(25) $$\begin{array}{c} O=\frac{l\,e\,n\,g\,t\,h\,\left(u\,n\,i\,o\,n\,\left(Q_{i},Q_{j}\right)\right)}{\min\left(l\,e\,n\,g\,t\,h\,\left(Q_{i}\right),l\,e\,n\,g\,t\,h\,\left(Q_{j}\right)\right)} \end{array}$$

where *Q*_*i*_ and *Q*_*j*_, respectively, represent the eigenvectors of the *i*th and *j*th results, *length*() represents the number of eigenvectors, and *union*() represents the union of the *i*th and *j*th results.

**FIGURE 3:**
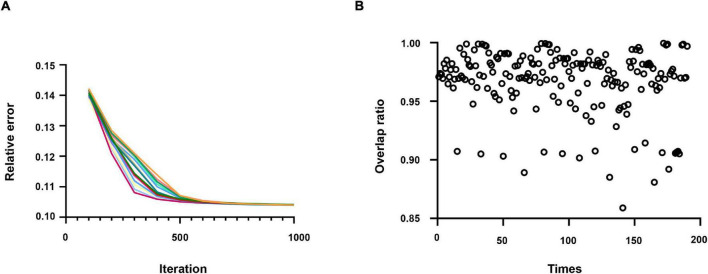
**(A)** The relative error of the results repeated running 20 times. Each time, the result is indicated by a different color. **(B)** The overlap rate of the results was repeated 20 times.

As displayed in Figure 3A, the relative error of the objective function only changes slightly after multiple runs of the algorithm. Moreover, in Figure 3B, each scatter represents the overlap rate of two results, and it can be found that the overlap rate of features repeated 20 times is more than 0.85. Hence, these pieces of evidence prove the robustness and repeatability of the SCNMF algorithm.

### Comparison With Classical Methods

**FIGURE 4:**
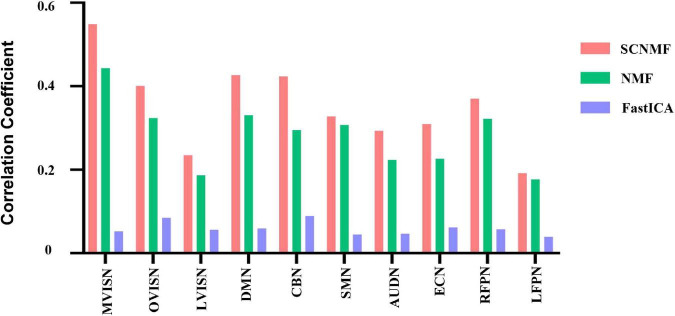
The proposed algorithm is compared with NMF and FastICA. SCNMF, NMF, and FastICA are used to extract large-scale brain network components, and the correlation coefficients between components and network templates were calculated, respectively.

To further verify the effectiveness of the SCNMF method proposed in this manuscript, the correlation analysis between the components obtained by the SCNMF, NMF, and FastICA methods and the ten resting-state network templates obtained by Smith was performed in this study. The experiment was repeated 20 times. Figure 4 shows the histograms of the correlation coefficients between the components calculated by SCNMF, NMF, and FastICA methods and the ten network templates, respectively. From Figure 4, it can be seen that among the ten brain networks, the correlations between the components obtained by SCNMF and the brain network templates were higher than those obtained by NMF and FastICA.

The generalized estimating equation method was used to investigate whether there was a statistical difference in the correlation coefficients between the brain network templates and brain network components obtained by the three methods. The results showed that there was a significant difference between the three methods(*p* < 0.001). Subsequently, paired *post hoc* analyses were performed for the three methods with Bonferroni correction for multiple comparisons, and the results are shown in Table 2. As can be seen from the table, there were significant differences between any two of the three methods. This indicates that the SCNMF method proposed by this study does help to improve the spatial source signal recovery in fMRI data analysis and better extract the region of interest from fMRI data compared with the classical NMF and FastICA methods.

**TABLE 2:** Multiple comparisons.

				95% confidence interval	
Method	Method	*df*	Sig	Lower bound	Upper Bound
SCNMF	NMF	1	*p* = 0.034	0.0052	0.1871
	FastICA	1	*p* < 0.001	0.2242	0.3795
NMF	FastICA	1	*p* < 0.001	0.1549	0.2563

### Selection of Window Width and Step Length

**FIGURE 5:**
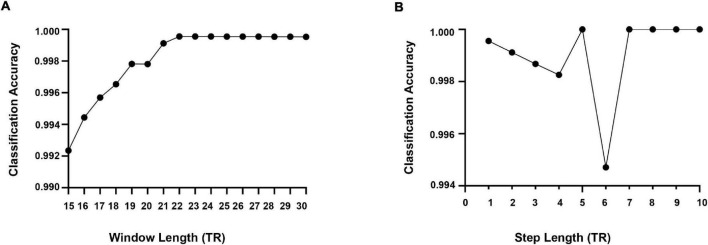
**(A)** The optimal window length is estimated based on the classification accuracy. **(B)** Estimation results of the optimal step length in terms of classification accuracy.

At present, there is no uniform regulation on the selection of time window length when using the sliding window method. According to previous studies, when using the sliding window for dynamic FC analysis, the cognitive state can be correctly identified when the window size is 30–60 s (Shirer et al., 2012). To more validly capture the dynamic pattern of psychiatric disorders and HCs, we conducted softmax classification for these four groups of subjects and determined the optimal window width through the highest classification rate. First, we calculated the correlation matrix of each subject between 15TR and 30TR, and the step size was 1TR. Each correlation matrix was triangulated to form a one-dimensional vector (45 × 1) as a classification feature. Repeat the above two steps to calculate the classification characteristics of all the subjects. Whereafter, the characteristics of these four subjects were classified by softmax. The ratio of training samples and test samples was 7:3. As displayed in Figure 5A, when the window width was 22TR, the classification rate was the highest. Therefore, we chose the optimal window width of 29TR for the dynamic FC of all subjects.

Meanwhile, we used the same softmax classification algorithm to determine the step size of the sliding time window and calculated the classification rate for step sizes from 1TR to 10TR. From Figure 5B, it can be seen that the classification rate was high when the step size is 5TR, 7TR, 8TR, 9TR, and 10TR, while referring to the results of existing studies, 5TR was chosen as the step size in this study.

### Dynamic Connectivity States

**FIGURE 6:**
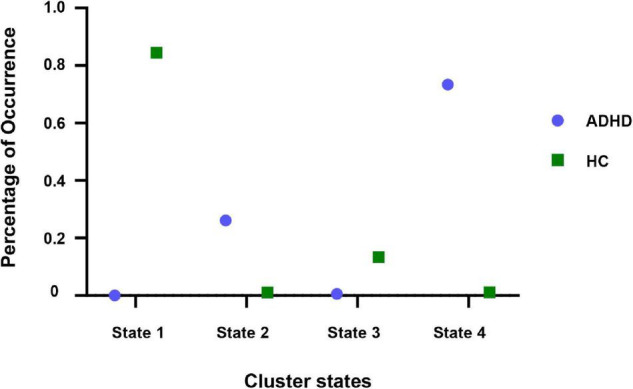
Percentage of recurring dynamic windows for adult ADHD and HCs in each state.

In this study, the k-means clustering method was used to cluster the dynamic connectivity matrix of all subjects defined as input samples. In addition, the elbow criterion was used to determine the optimal clustering performance, i.e., the number of clusters was set to 4. By k-means clustering on account of the dynamic FC window, four states that appear repeatedly in the whole scanning process and across subjects were obtained. The specific distribution of adult ADHD and HCs dynamic windows in different states is shown in Figure 6. As can be seen from Figure 6, adult ADHD was mainly distributed in state 2 and state 4, and HC subjects were concentrated in state 1 and state 3. Interestingly, each state had a representative subject that accounted for a large proportion of subjects, with 84.47% of HC subjects in state 1, 26.13% of ADHD subjects in state 2, 13.38% of HC subjects in state 3, and 73.36% of ADHD subjects in state 4.

**FIGURE 7:**
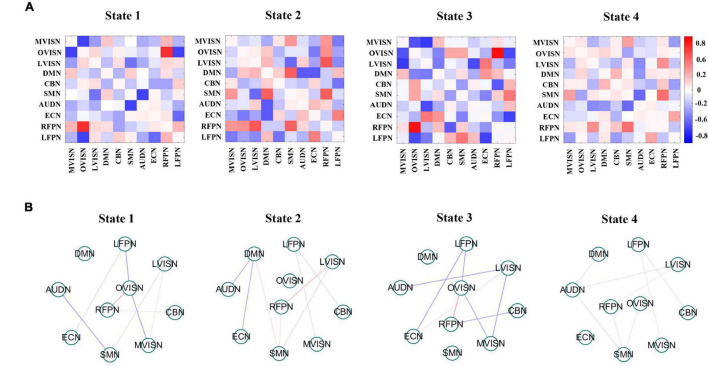
**(A)** Group-specific centroids of dynamic functional connectivity (FC) states for adult Attention-deficit/hyperactivity disorder (ADHD) and healthy controls (HCs). Dynamic FC states stemmed from k-means clustering employing the sliding window approach. **(B)** It shows that the absolute strength of dynamic FC of large-scale networks in each state is at the top 15%. The red line expresses positive connectivity between networks, and the blue line expresses minus connectivity between networks. The thickness of the line indicates the strength of connectivity.

What is more, Figure 7A shows the four dynamic FC states for adult ADHD and HCs. Each state represented the centroid of a state and presumptively reflected a connectivity state steadily presented in the fMRI data. A distinctive feature was that the overall connectivity of state 1 to state 4 was different in terms of the strength of the connectivities between the functional networks. Specifically, state 2 and state 3 were stronger connected states, and state 1 and state 4 were weaker connected states. It was worth noting that the weaker connectivity state was easier to detect than the stronger connectivity state, which meant that the connectivity of the brain network mainly depended on the comparatively weaker connectivity state.

To better visualize the connectivity patterns of the large-scale network in different states, Figure 7B displays the top 15% of the absolute value of the connectivity strength in each state. In states 1 and 3, the large-scale network exhibited connectivity characteristics around LVISN and OVISN, and also involved RFPN. Furthermore, the connectivity between SMN and RFRN was positively connected in both states. On the whole, the connectivity distribution was more complex. In state 2, the large-scale network presented connectivity characteristics around DMN and RFPN. In state 4, the overall connectivity strength was weaker. At the same time, the overall connectivity strength of adult ADHD patients was lower than that of HCs, indicating that the connectivities between large-scale networks of adult ADHD subjects were interrupted or decreased. In addition, this illustrates that the SCNMF algorithm can be effective in detecting patterns of connectivity specific to the large-scale networks of adult ADHD patients and HCs.

**FIGURE 8:**
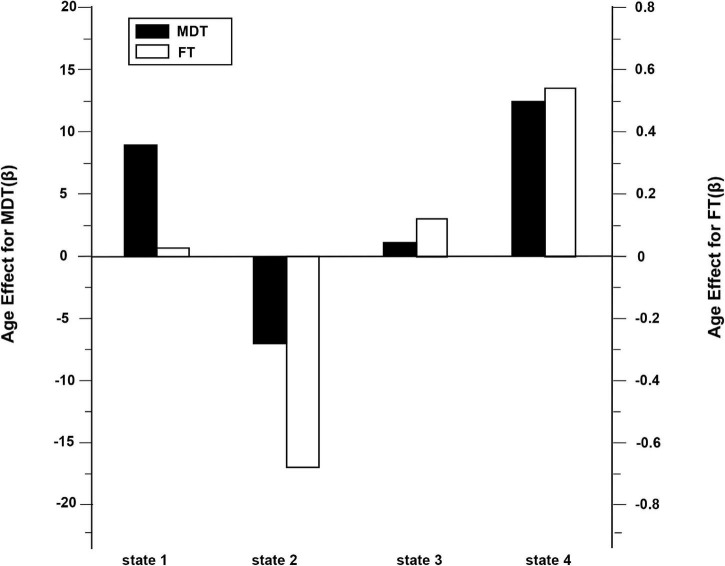
The mean dwell time (MDT) and fraction of time (FT) from four states varied with age. The positive coefficient of β manifests that the older subjects expend more time in the homologous state. Simultaneously, the subtractive coefficient of β manifests that the younger subjects expend more time in that state.

To explore how different quantitative summary metrics of dynamic FC vary with age, the MDT for each state is statistically analyzed employing univariate linear regression, and the results are shown in Figure 8. The parameter β stands for the linear regression coefficient. A positive coefficient of β expresses that the older subjects expended more time in the commensurable state whereas a subtractive coefficient of β indicated that younger subjects expended more time in that state.

As can be seen in Figure 8, older subjects had longer MDT in state 1, state 3, and state 4. In contrast, younger subjects showed longer MDT in state 2. Since adult ADHD was mainly distributed in states 2 and 4, it can be seen that older adults with ADHD were mainly in state 4 and younger adults with ADHD were mainly in state 2. However, the effect of age was smaller for HC subjects.

**FIGURE 9:**
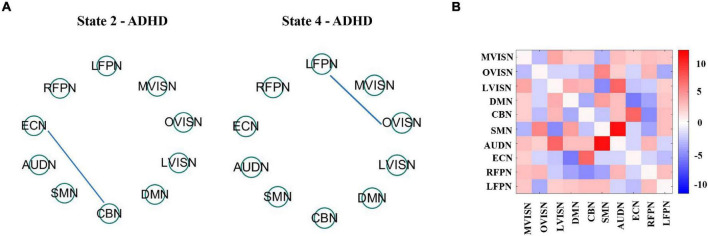
**(A)** It shows age association across the dynamic FC state 2 and state 4. The blue line expresses a negative relevance among FC and age. All of the outcomes expressed correspond to the false discovery rate (FDR) multiple comparison correction threshold of *p* < 0.05. **(B)** State difference between dynamic FC state 2 and state 4 is obtained using paired *t*-test with the FDR threshold (*p* < 0.05). State variation was visualized by drawing the log of *p* value with the sign of *t* statistics, −*sign*(*t*)log(*p*).

To further research the influence of age on adult ADHD in states 2 and 4, the median participant in each state was calculated employing the window correlation matrix of each subject. It has been documented that the median matrix can be deemed a representative model of the subjects in a specific state (Damaraju et al., 2014). Subsequently, univariate analysis was applied to research the correlation between connectivity and age in the median matrix of these subjects, as shown in Figure 9A.

The effect of age on the large-scale network connectivity of adult ADHD subjects in state 2 and state 4 was only reflected in a few large-scale network connectivities. However, the effects of age on the two states were different. Specifically, in state 2, the negative age-related FC was between CBN and ECN. In state 4, age only affects the FC between OVISN and LFPN.

Then, to assess adult ADHD state differences in dynamic functional connectivity between state 2 and state 4, we used the median of these subjects and applied paired *t*-test to assess state differences. All outcomes were consistent with the false discovery rate (FDR) multiple comparison correction threshold *p* < 0.05. The state modification was visualized by drawing the log of *p* value with the sign of *t* statistics, −*sign*(*t*)log(*p*). The momentous state difference between functional networks is shown in Figure 9B. The results showed that FC in adult ADHD was significantly different under these two states. Among them, the positively significant differences in brain functional connectivity were mainly between ECN and CBN, and between AUDN and SMN. Relatively speaking, the FCs with negatively significant differences were mainly between LVISN and SMN. These pieces of evidence indicate that large-scale network connectivities in adult ADHD have commonality and specificity and may serve as a biomarker for the study of adult ADHD in the future.

## Discussion

In this study, we proposed a new spatial constrained NMF method based on the authentic reference signal, which mined available spatial prior information from the group fMRI data and amalgamated this prior information into the NMF method. By comparing with other classical methods, the SCNMF algorithm proposed in this manuscript can better extract the region of interest. On this basis, this study proposed a new analytical framework for assessing large-scale functional network changes in adult ADHD and healthy subjects, exploring the differences in dynamic functional connectivity patterns between the two groups. To our knowledge, this is the first time that NMF has been integrated into the dynamic functional connectivity of large-scale networks and applied to adult ADHD.

So far, the most common dynamic functional connectivity method for fMRI data is ICA. The ICA method assumes independence between components to obtain spatial components, but there is a problem that it cannot extract sparse sources efficiently. The NMF method can be a good solution to this problem because it can decompose the data into a linear combination of sparse, partial-based non-negative features. In addition, it has been shown that adding prior information to NMF can improve its analytical performance on fMRI data. Therefore, this study proposed an SCNMF method based on the real information of fMRI data. The proposed SCNMF method was quantitatively compared with the traditional methods NMF and FastICA, and the results showed that the spatial brain networks detected by SCNMF were better than those detected by NMF and FastICA. From this point of view, it seems that the SCNMF method does help to improve the recovery of spatial source signals in fMRI data analysis, while the extracted brain networks of interest have higher separation quality and accuracy.

Dynamic FC collared steady connectivity patterns, which are not watched in stationary FC. The FC of the brain is not static but changed over time. Therefore, more valuable information can be discovered by observing the differences in intergroup connectivity captured by dynamic states. According to the results of this study, the differences between ADHD and HCs were not limited to a single dynamic FC state but distributed in four dynamic FC states.

The ECN consists of the cingulate cortex, prefrontal cortex, insular cortex, and striatum and is involved in cognition, inhibitory behavior, emotion, and pain (Smith and Nichols, 2009). It is also referred to as the “transitional network connecting cognition and emotion/sensation” (Laird et al., 2011). The medial prefrontal cortex is an important component of the ECN and is an area known to be associated with information processing. In most psychiatric disorders, coordination between executive function and internal and external attention is considered to be severely impaired. Interestingly, ADHD patients had elevated connectivity between the ECN and RFPN in the dynamic functional connectivity state compared to HCs. Furthermore, it had been shown that ADHD abnormalities were strongly associated with the ECN (Makris et al., 2009; Bush, 2010; Posner et al., 2014). Therefore, the unbalanced connectivity between ECN and FPN may help to explain the broader psychopathological features of ECN abnormalities in adult ADHD.

Another important finding of dynamic FC between large-scale networks was that adult ADHD had enhanced FC between ECN and CBN compared to HCs, but connectivity became smaller with age. According to a literature review, ADHD patients had demonstrated stronger internal connectivity in the CBN (Mostert et al., 2016). In addition, an increased FC in the ECN and the anterior cingulate cortex of the cerebellum was previously found in ADHD (McCarthy et al., 2013; Wang et al., 2013), which was consistent with our findings. However, our results complemented existing studies and found out the effects of age on ADHD.

Cortese et al. conducted a meta-analysis of 55 articles (39 for children and 16 for adults), concentrating on clinical features or particular neuropsychological structures in ADHD. They discovered that adult ADHD hypoactivation was prominent in the frontoparietal networks, while adult ADHD hyperactivation appeared in the visual, dorsal attention, and DMN (Cortese et al., 2012). In the present study, adult ADHD had enhanced connectivity in the DMN and SMN relative to HCs. Interestingly, adult ADHD also had enhanced RFPN and SMN connectivity. Therefore, there was a great difference between the strength of the FC within the network and the FC between the networks. We can take this as a starting point and verify it in the future research center.

It is worth mentioning that there is an age-related effect of MDT in dynamic states in adult ADHD and HCs. Specifically, it was observed that older ADHD had a longer dwell time in the relatively weaker connected state 4, whereas younger ADHD had a shorter dwell time in the relatively stronger connected state 2. However, age had little effect on HCs. This is slightly different from the adult connectivity model previously found in adult studies (Fair et al., 2008; Zuo et al., 2010), suggesting that neurodevelopment in ADHD differs from that of HCs. In addition, the difference in results may also be due to differences in the scale of the study. In this manuscript, the entry point of the study is the brain network, while some literature has considered brain regions, and the difference in scale may also affect the final results. In the future, we can consider more comprehensive studies from multiple scales to further discover the pathogenesis of ADHD.

In addition, there was almost no overlap in the four states obtained in dynamic functional connectivity with only one type of subjects in each state. Considering that it is possible that this is due to a problem with the data set, for this reason, we changed the data set for validation. Forty-five HCs and 40 adult ADHD subjects were downloaded from openfMRI. Ten cognitive networks were extracted using the algorithm proposed in this manuscript, followed by dynamic functional connectivity analysis. The four dynamic functional state distributions obtained were similar to the results obtained in this manuscript.

## Data Availability Statement

The original contributions presented in the study are included in the article/supplementary material, further inquiries can be directed to the corresponding author.

## Author Contributions

YL and WZ conceived and designed the study. YL performed the experiments and wrote the manuscript. YL, JY, and WN collected the samples and analyzed the data. JD, YS, and SL reviewed and edited the manuscript. All authors read and approved the final version of the manuscript.

## Conflict of Interest

The authors declare that the research was conducted in the absence of any commercial or financial relationships that could be construed as a potential conflict of interest.

## Publisher’s Note

All claims expressed in this article are solely those of the authors and do not necessarily represent those of their affiliated organizations, or those of the publisher, the editors and the reviewers. Any product that may be evaluated in this article, or claim that may be made by its manufacturer, is not guaranteed or endorsed by the publisher.

## Footnotes

1
http://www.nitrc.org/projects/fcon_1000/
